# Transcriptome analysis of the *Cryptocaryon irritans *tomont stage identifies potential genes for the detection and control of cryptocaryonosis

**DOI:** 10.1186/1471-2164-11-76

**Published:** 2010-01-29

**Authors:** Yogeswaran Lokanathan, Adura Mohd-Adnan, Kiew-Lian Wan, Sheila Nathan

**Affiliations:** 1 School of Biosciences and Biotechnology, Faculty of Science and Technology, Universiti Kebangsaan Malaysia, Selangor, Malaysia; 2 Malaysia Genome Institute, UKM-MTDC Technology Centre, Selangor, Malaysia

## Abstract

**Background:**

*Cryptocaryon irritans *is a parasitic ciliate that causes cryptocaryonosis (white spot disease) in marine fish. Diagnosis of cryptocaryonosis often depends on the appearance of white spots on the surface of the fish, which are usually visible only during later stages of the disease. Identifying suitable biomarkers of this parasite would aid the development of diagnostic tools and control strategies for *C. irritans*. The *C. irritans *genome is virtually unexplored; therefore, we generated and analyzed expressed sequence tags (ESTs) of the parasite to identify genes that encode for surface proteins, excretory/secretory proteins and repeat-containing proteins.

**Results:**

ESTs were generated from a cDNA library of *C. irritans *tomonts isolated from infected Asian sea bass, *Lates calcarifer*. Clustering of the 5356 ESTs produced 2659 unique transcripts (UTs) containing 1989 singletons and 670 consensi. BLAST analysis showed that 74% of the UTs had significant similarity (E-value < 10^-5^) to sequences that are currently available in the GenBank database, with more than 15% of the significant hits showing unknown function. Forty percent of the UTs had significant similarity to ciliates from the genera *Tetrahymena *and *Paramecium*. Comparative gene family analysis with related taxa showed that many protein families are conserved among the protozoans. Based on gene ontology annotation, functional groups were successfully assigned to 790 UTs. Genes encoding excretory/secretory proteins and membrane and membrane-associated proteins were identified because these proteins often function as antigens and are good antibody targets. A total of 481 UTs were classified as encoding membrane proteins, 54 were classified as encoding for membrane-bound proteins, and 155 were found to contain excretory/secretory protein-coding sequences. Amino acid repeat-containing proteins and GPI-anchored proteins were also identified as potential candidates for the development of diagnostic and control strategies for *C. irritans*.

**Conclusions:**

We successfully discovered and examined a large portion of the previously unexplored *C. irritans *transcriptome and identified potential genes for the development and validation of diagnostic and control strategies for cryptocaryonosis.

## Background

The ciliate protozoan *Cryptocaryon irritans *(Family: Cryptocaryonidae) [1] is an obligate ectoparasite that causes cryptocaryonosis, also known as white spot disease, in marine fish [2]. Although *C. irritans *is commonly found in tropical, subtropical and warm temperate waters at low infection intensity [3], infection by this parasite has emerged as a major problem in confined surroundings such as in mariculture and aquariums [4,5] due to the buildup of the parasite and high population density of fish in these systems [6].

*C. irritans *penetrates the skin, gills and eyes of the fish and impairs the functioning of these organs. The key signs of cryptocaryonosis are the formation of pinhead-sized whitish nodules, mucus hyperproduction, skin discoloration, anorexia and respiratory difficulties [2]. *C. irritans *has low host specificity and can infect a taxonomically broad host range, including both temperate marine fish and saltwater-adapted fresh-water fish that do not encounter the disease naturally [7,8].

The *C. irritans *life cycle involves four stages that require a mean time of 1-2 weeks for completion independent of an intermediate host [2]. The parasitic stage trophont burrows itself within the host epithelium and feeds on both tissue debris and body fluids. During this period, the whitish nodules are observed on the body and fins, depending on the severity of the infection. The mature trophonts leave the host as protomonts after 3-7 days. The protomonts sink and adhere to the substratum following which they encyst and enter the reproductive stage. These newly formed tomonts undergo a sequence of asymmetric binary fissions to become daughter tomites inside the cyst wall. Between days 3-72, cyst rupture leads to the asynchronous release of differentiated tomites into the environment as theronts. A tomont produces approximately 200 theronts, and this infective stage parasite swims freely to find a host and rapidly penetrates the host epidermal layer. The infectivity of theronts decreases 6-8 h post-excystment [2,5].

To date, no commercial vaccines, drugs or diagnostic kits have been developed for white spot disease. Control of this parasite is hindered by factors such as the embedment of the parasite in the host epithelium, which renders many chemicals ineffective; asynchrony in theront release and trophont exit; and ineffectiveness of chemical treatment in large-volume systems [2]. In addition, lack of parasite genomic data has hampered the use of molecular tools in developing control strategies for *C. irritans*.

Many parasites are phylogenetically distant organisms, and the application of genetic tools to solve important parasite-related biological problems has been slow due to the limitations in gene identification by heterologous probing and lack of genomic studies [9]. Expressed sequence tag (EST) analysis of parasites can provide a vast amount of genomic data that can serve as an important resource for transcriptome exploration including gene discovery, gene structure identification, genome annotation and identification of potential molecules for drug and vaccine development [10,11]. EST analysis is also an efficient method of identifying differentially expressed genes at different developmental stages. Currently 33 *C. irritans *nucleotide sequences are known, but no EST records are available for these in the National Center for Biotechnology Information (NCBI) database.

In this study, we constructed a cDNA library of *C. irritans *tomonts to generate ESTs. The Asian sea bass (*Lates calcarifer*) was selected as the host because this species is important in commercial aquaculture and fisheries in the Asia-Pacific region, and is exposed to cryptocaryonosis. By analyzing the ESTs generated, we could predict transmembrane regions, glycosylphosphatidylinositol (GPI) anchor signals, signal peptides, and amino acid repeats, and this helped in identifying proteins that could be useful in developing disease control strategies. These data provide a foundation for further studies on both the *C. irritans *genome and proteome that would lead to a better understanding of the pathogenicity of this organism.

## Methods

### Parasite Isolation

*C. irritans *tomonts were collected from infected adult *L. calcarifer *(340-440 g) obtained from a sea cage culture facility at Bukit Tambun, Penang, Malaysia. The fishes were reared in 150 L aquariums filled with 100 L of seawater at a salinity of 30 ppt. The disease was induced by placing ice bags inside the aquariums twice a day, which lowered the water temperature from 28°C to 19°C. Glass Petri dishes were placed at the bottom of the aquarium once the white spots were visible to the naked eye. The following day, the Petri dishes were collected and replaced with new ones. The adhering tomonts were gently scrapped from the Petri dishes into a cavity block. All tomonts were cleaned with autoclaved seawater, transferred to microcentrifuge tubes, snap-frozen in liquid nitrogen and stored at -80°C until further used.

### RNA isolation

Total RNA was isolated from tomonts using TRI Reagent^® ^(Molecular Research Center, Inc., USA). TRI Reagent^® ^was added to the frozen tomonts, and the mixture was then mashed with a plastic mini-pestle until the material was completely homogenized. The subsequent steps were performed according to the manufacturer's protocol. The total RNA was resuspended in TE buffer (pH 7.4) and the quantity and quality of the RNA aliquots were checked on a bioanalyzer (Agilent Technologies). mRNA was isolated from good quality total RNA using the Illustra™ mRNA Purification Kit (GE Healthcare, UK).

### cDNA library construction

A cDNA library of *C. irritans *tomonts was constructed using the ZAP-cDNA Library Construction Kit (Stratagene, USA). Briefly, mRNA was reverse transcribed into cDNA and size-fractionated cDNA was inserted into the Uni-Zap λ vector in a sense orientation. The recombinant λ vector was subsequently packaged into lambda particles, transfected into XL1-Blue MRF' cells, and plated on agar with X-gal and isopropyl-1-thio-β-D-galactopyranoside (IPTG). The primary library was amplified to obtain a stable secondary library with a higher titer.

### Plasmid extraction and sequencing

Aliquots of the secondary library were subjected to *in vivo *mass excision, and the excised plasmids from randomly selected clones were extracted using the Montage™ Plasmid Miniprep_96 _Kit (Millipore, USA). The inserts were sequenced from the 5' end using the SK primer and the BigDye^® ^Terminator v3.1 Cycle Sequencing Kit (Applied Biosystems Inc., USA). The ABI PRISM 3730xl DNA Analyzer (Applied Biosystems Inc., USA) was used for sequencing.

### Sequence Analysis

Sequences were subjected to Phred [12,13] analysis with a cut-off quality value (QV) of 20. Vector sequences were trimmed using Cross_match [14] and StackPACK version 2.2 [15] was used to cluster the EST data. The resulting unique transcripts (UTs) were compared with the nonredundant (nr) Genbank nucleotide and protein databases at the National Center for Biotechnology Information (NCBI) site using TBLASTX and BLASTX [16], respectively.

The ESTs of *Ichthyophthirius multifiliis *were downloaded from dbEST at NCBI, and BLASTN analysis was performed to compare *I. multifiliis *ESTs with the UTs obtained in this study. The *C. irritans *ESTs were further translated using the Ciliate, Dasycladacean and Hexamita Nuclear Code, and BLASTX was used to compare these to protein sequences of *Tetrahymena thermophila *obtained from the nr protein database (NCBI) andthose of *Plasmodium falciparum *obtained from PlasmoDB 5.5 [17]. The cut-off E-value was set to <10^-5 ^in all BLAST analyses.

Further comparisons were made to conserved protein families by comparing the Pfam [18] protein family and SUPERFAMILY [19] protein superfamily assignments of *C. irritans*, *T. thermophila*, and *P. falciparum*. The protein domain assignments for *C. irritans *were derived from the InterProScan results using BLAST2GO [20,21]. The Pfam protein families for *P. falciparum *3D7 and *T. thermophila *were obtained from the *P. falciparum *3D7 directory at the *Plasmodium falciparum *Genome Project FTP server [22] and *Tetrahymena *Genome Database FTP server [23], respectively. The SUPERFAMILY domain assignments for *T. thermophila *and *P. falciparum *were obtained from SUPERFAMILY Assignments for Genomes and Sequence Collections [24].

Simple sequence repeats (SSRs) in the nucleotide sequences were identified using the MIcroSAtellite identification tool (MISA) [25]. The poly-A and poly-T sequences at the terminal regions of the UTs were removed before SSR identification.

The translation codes of ciliates differ from the standard translation codes; therefore, all nucleotide sequences were translated to peptide sequences prior to further analysis. Virtual Ribosome [26] was used to translate the nucleotide sequence to peptide sequences taking ciliate translation codes into consideration. The parameters were set such that all sequences were treated as partial sequences, and the presence of a start codon was not essential for starting a coding sequence (CDS); this aided the recognition of partial CDSs.

Gene ontology (GO) annotations were performed using Blast2GO [20]. The peptide sequence was loaded into the Blast2GO program, and BLASTP with a minimum E-value of < 10^-5 ^was performed by the program prior to mapping and annotation into GO terms. In addition, the UTs were annotated according to the Kyoto Encyclopedia of Genes and Genomes (KEGG) [27] orthology (KO) by the KEGG Automatic Annotation Server (KAAS) [28] and pathways of the annotated UTs KO terms were identified using the KO Based Annotation System (KOBAS) server [29]. The peptide sequences of translated UTs were used as the query sequence, and the bi-directional best hit (BBH) method was employed to obtain the KO terms for the query sequences. The KO list was then loaded into the pathway identification tool at the KOBAS web-server to identify statistically augmented pathways in the data set [29]. The entire *T. thermophila *gene set was used for background distribution. Significantly enriched pathways were considered to be those with P < 0.05 from binomial tests performed on the KOBAS server [29,30].

Putative membrane proteins were identified by SignalP 3.0 [31], Localizome [32], ProtCOMP 6.1 [33], TMHMM 2.0 [34] or Sosui 1.1 [35]. Putative GPI-anchored proteins were predicted using GPI-SOM [36], Big-π [37] and FragAnchor [38]. GPI-SOM predicts both the N-terminal signal peptide and C-terminal GPI-anchor signal whereas Big-π and FragAnchor only predict the C-terminal GPI-anchor signal. The repeats in the UTs were identified using Reptile [39] and RepSeq [40].

## Results

### Sample collection and cDNA library construction

White spots were observed on the fish body 3 days after the arrival from the sea cage culture. The fishes harbored low levels of *C. irritans *infection when brought in from the sea cage and became stressed due to the frequent and drastic temperature fluctuations. This lowered their immunity and resulted in the outbreak of white spot disease [41]. Total RNA was prepared from the harvested tomonts, and Bioanalyzer analysis confirmed that the RNA integrity was within the acceptable range (5.9 to 6.3). mRNA was isolated from the total RNA and used as the template for cDNA synthesis. The cDNA was size-fractionated to select for cDNA strands longer than 400 bp prior to construction of the cDNA library. The constructed primary library of tomont cDNA had a titer of 1.28 × 10^6 ^pfu. X-Gal/IPTG screening indicated a recombination efficiency of 93% while PCR amplification of 96 random clones showed that the insert sizes ranged from 1-4 kb with an average size of 1.3 kb.

### EST sequencing and analysis

A total of 5760 clones were selected for plasmid extraction and subsequent single-pass sequencing from the 5' end. After eliminating low-quality, vector-contaminated and very low complexity sequences as well as those of length less than 50 bp, 5356 (93%) good-quality sequences were obtained for further analysis. The sequences were loaded into a command line version of StackPACK V2.2. Subsequently, clustering with d2_cluster, alignment using PHRAP, and assembly analysis using stack_Analyze and CRAW were performed [42]. This resulted in the identification of 2659 UTs consisting of 670 consensi from 3367 sequences and 1989 singletons (Table 1).

**Table 1:** Summary of *C. irritans *EST analysis

	Number (percentage)
Total number of clones sequenced	5760
Number of high quality sequences	5356 (93)
Number of consensi	670
Singletons	1989
Unique transcripts (UTs)	2659
Number of known genes	1966 (74)
Number of unknown genes	692 (26)

Seventy five percent (1989/2659) of the UTs were singletons, which precluded the need to normalize or subtract the library generated in this study for data mining and transcriptome survey. The genes expressed at the tomont stage of development were mined and the gene discovery rate (percentage of unique sequences over total sequences analyzed) was 50%, which is acceptable for a non-normalized library. The assembled ESTs showed that some genes were expressed at very high levels, as much as 2.5% of all the expressed transcripts at the tomont stage. The 20 most abundant genes at the tomont stage are listed in Table 2. The BLASTX results revealed that the sequences of cn48 and cn10, two highly expressed UTs, were similar to sequences from bacteria. These UTs could be derived from new genes that have not yet been identified in other lower eukaryotes and could have possible functions that are not related to the BLASTX feedback. These UTs demonstrated no hits in InterProScan as well in the BLASTN analyses with both the dbEST and nr nucleotide collection databases in Genbank; therefore, the functions of these highly expressed unique proteins should be determined experimentally. Most of the other highly expressed UTs had corresponding homologs in other ciliates and protozoa, and several UTs were highly similar to other protozoan genes (Table 2).

**Table 2:** The 20 most abundantly encountered genes at the tomont stage

UT ID	No. ESTs	Putative Identity	Organism	% Identity	E Value
cn48	136	Outer membrane adhesin like protein	*Prosthecochloris vibrioformis*	30.95	4E-09
cn52	98	28S ribosomal RNA	*Ichthyophthirius multifiliis*	84.00	0
cn10	79	RTX toxins and related Ca^2+^-binding proteins	*Magnetospirillum magnetotacticum*	34.78	3E-09
cn42	68	Agglutination/immobilization antigen isoform 1	*Cryptocaryon irritans*	71.82	1E-118
cn24	59	Insect antifreeze protein	*Tetrahymena thermophila*	22.67	1E-38
cn1	54	Granule tip protein 2	*Tetrahymena thermophila*	23.24	7E-08
cn21	50	No significant hit			
cn57	48	Agglutination/immobilization antigen isoform 1	*Cryptocaryon irritans*	41.04	7E-58
cn107	40	Polyubiquitin	*Plasmodium falciparum*	97.21	0
cn123	37	Hypothetical 18K mitochondrion protein	*Carassius auratus*	49.05	3E-15
cn41	35	Agglutination/immobilization antigen isoform 1	*Cryptocaryon irritans*	66.87	1E-119
cn14	34	Beta-glucanase/Beta-glucan synthetase	*Hahella chejuensis*	37.95	5E-20
cn25	32	MCM2/3/5 family protein	*Tetrahymena thermophila*	51.06	0
cn11	29	Tubulin beta chain	*Tetrahymena thermophila*	95.11	0
cn174	28	ER-type hsp70	*Paramecium tetraurelia*	74.88	0
cn81	23	Chitinase	*Kurthia zopfii*	32.03	3E-47
cn66	22	Insect antifreeze protein	*Tetrahymena thermophila*	23.49	2E-24
cn110	22	Calpain-like protein	*Sterkiella histriomuscorum*	32.95	8E-29
cn56	20	Agglutination/immobilization antigen isoform 4	*Cryptocaryon irritans*	42.67	4E-65
cn80	20	Tubulin/FtsZ family, GTPase domain containing protein	*Tetrahymena thermophila*	93.63	0

**Figure 1:**
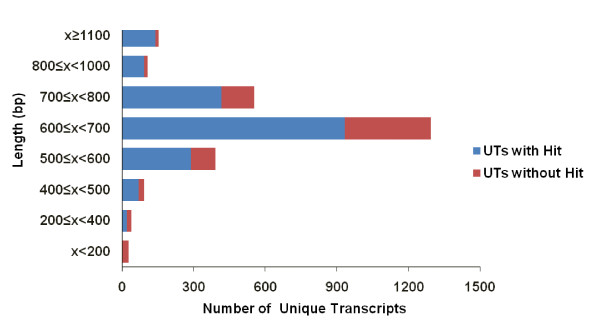
**Length of unique transcripts versus BLAST hit**. The relationship between the length of the unique transcripts (UTs) and the number of UTs with BLAST hits.

The BLASTX similarity search showed that 72% (1909) of the UTs had significant matches with sequences in the NCBI nr protein database (Additional File 1). Among the 1909 matches, 298 matches were to genes of unknown function. Most of these matches were to hypothetical proteins of *T. thermophila *SB210 and *Paramecium tetraurelia strain *d4-2. Subsequent comparison by TBLASTX showed that another 57 of the 750 UTs with no matches from the BLASTX analysis exhibited significant matches to sequences in the NCBI nr nucleotide database. A plot of the UTs length versus the number of BLAST hits (Figure 1) showed that more than 96% (668/693) of the UTs without hits had a length of more than 200 bp. Therefore, we presume that the reason why most UTs do not have significant hits is not because these are short but because these are novel sequences that are specific to the *C. irritans *transcriptome. Exceptions could possible arise from possible genomic DNA contamination and the presence of very long untranslated regions and noncoding RNAs. However, there is also the possibility that some of these novel genes may exist in other previously sequenced organisms but have never been expressed or captured for sequencing.

**Figure 2:**
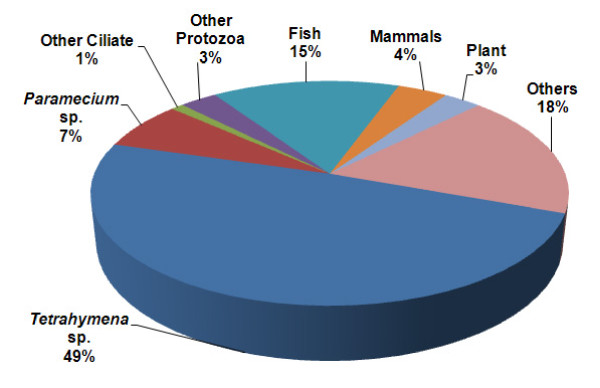
**Distribution of best BLASTX matches according to organisms**. A diagrammatic representation of organism distribution according to UTs with the best match to the NCBI non-redundant protein database.

**Figure 3:**
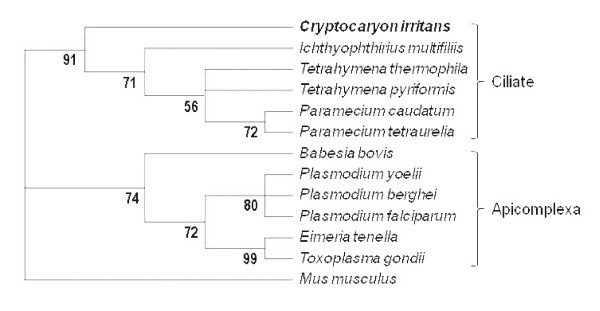
**Phylogenetic analysis of *C. irritans***. A maximum parsimony tree inferred from the complete β-tubulin amino acid sequence of *C. irritans *and other alveolates, with *Mus musculus *as an outgroup. The *I. multifiliis *β-tubulin amino acid sequence was inferred from the EST dataset. The numbers below branches are the bootstrap values of 1000 iterations of the data file.

Organism distribution based on the BLASTX analysis results is shown in Figure 2. A total of 57% of the matches were to other ciliate species, mainly to *T. thermophila *SB210 and *P. tetraurelia *strain d4-2. Consistent with this, the phylogenetic analysis of β-tubulin sequences supported previously described taxonomic relationships associating *C. irritans *with other ciliates such as *I. multifiliis*, *T. thermophila *and *P. tetraurelia *in a distinct cluster and more distantly than other nonciliate protozoa such as *Plasmodium sp.*(Figure 3) [1,43,44]. Another 15% of the matches were with fish sequences, which might be due to host contamination in the parasite sample because the sample was collected after *in vivo *propagation.

### Comparative analysis with *I. multifiliis*

*I. multifiliis *is the fresh-water counterpart of *C. irritans *that causes white spot disease in fresh-water fishes. Although both *C. irritans *and *I. multifiliis *share many external features and a parallel life cycle, ultrastructural and taxonomic studies have concluded that these parasites are distantly related and that their striking similarities are a result of convergent evolution [1,45]. A BLASTN search against 33 516 redundant *I. multifiliis *ESTs showed that 260 UTs of *C. irritans *have significant similarities with *I. multifiliis *ESTs. Among the 260 hits, 2 UTs had matches with *I. multifiliis *ESTs with low E values (10^-7 ^and 10^-9 ^respectively) and another 27 hits had an alignment percentages of more than 50%. Almost all of the 258 *C. irritans *UTs that had matches with the *I. multifiliis *ESTs also had matches with the sequences of other organisms with a higher E-value especially with *T. thermophila *and *P. tetraurelia *sequences. These EST matches can be assumed to present ESTs-encoding genes that are conserved in ciliates and are not exclusively present in these two parasitic ciliates. It is noteworthy that cn52, which was the second most abundant encountered consensus sequence, showed a high similarity to the highly abundant transcripts detected by *I. multifiliis *EST sequencing. Further BLASTN analysis showed that cn52 is highly similar to the *I. multifiliis *28S ribosomal RNA gene (GenBank accession number: EU185635.1) and to ribosomal RNA of other organisms (Table 2). Polyadenylation of *C. irritans *rRNA remains to be confirmed because it was recently discovered that the 28S rRNA of *I. multifiliis *was not only polyadenylated at the 3' end of the rRNA but also contained three extra internal polyadenylation sites [46].

### Comparative BLASTX analysis with *T. thermophila *and *Plasmodium falciparum*

**Figure 4:**
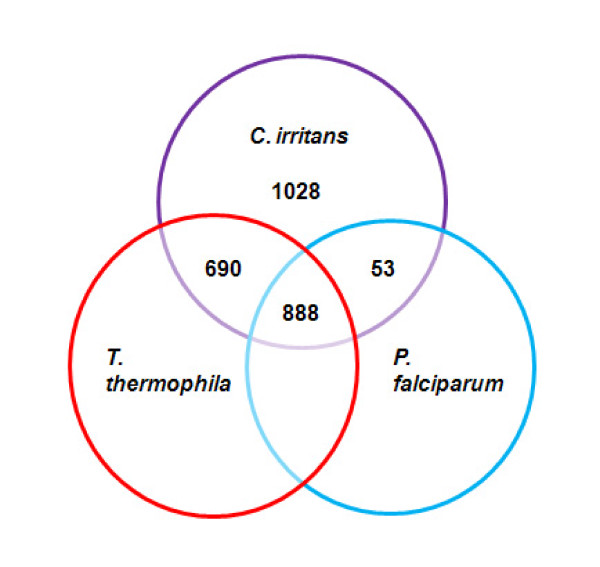
**Proteins shared among *C. irritans*, *T. thermophila *and *P. falciparum***. Venn diagram summary of *C. irritans *translated UTs comparison with *T. thermophila *and *P. falciparum *peptide sequences. The numbers at the overlapping area represent matching peptides (BLASTX of E < 10^-5^) in the relevant organism with the query 2659 translated UTs of *C. irritans*.

The *T. thermophila *genome and transcriptome sequences are publicly available, whilst *P. falciparum *is a protozoan parasite for which abundant biological information is readily available. BLASTX analysis of *C. irritans *UTs against *T. thermophila *and *P. falciparum *proteins was performed to identify homologous proteins in these organisms. The results are summarized in Additional File 2 and presented in Figure 4. A total of 1578 (59%) *C. irritans *UTs had significant similarity to *T. thermophila *proteins including 1156 UTs that had matches with annotated proteins of *T. thermophila*. In addition, 941 (35%) UTs showed significant similarity to *P. falciparum *proteins, including 748 UTs with matches to annotated proteins of *P. falciparum*. We identified 53 proteins that are similar to *P. falciparum *proteins but did not obtain significant hits with any *T. thermophila *proteins. In total, 888 (33%) UTs showed similarity to both *T. thermophila *and *P. falciparum *proteins.

### Comparative gene family analysis

The gene families of *C*. *irritans *were compared to those of *T. thermophila *and *P. falciparum *to characterize the conserved and unique proteins in these protozoans. The SUPERFAMILY domains are classified based on structural similarity whereas the Pfam domains were classified based on sequence similarity. The comparison statistics of the protein family and superfamily assignments of the three protozoans are presented in Table 3, and the complete table with the protein family and superfamily assignments according to the SUPERFAMILY and Pfam domains is shown in Additional File 3. The analysis showed that there is a large difference between *C. irritans *and the two other organisms in terms of the number of SUPERFAMILY and Pfam domains found. A total of 250 SUPERFAMILY domains and 345 Pfam domains were found to be present in all the three protozoan's, indicating that 77% of the *C. irritans *SUPERFAMILY domains and 57% of the Pfam domains are conserved in these organisms. In addition, 87% of *C. irritans *protein families are well conserved in *T. thermophila*. Interestingly, 28 SUPERFAMILY domains and 66 Pfam domains were found to be present exclusively in *C. irritans*. These included various domains of metabolic enzymes such as serine-threonine phosphatase, polysaccharide deacetylase, and oxidoreductase and domains of structural proteins such as axonemal dynein light chain, proteasome subunit A, conserved membrane protein, and ligand-binding domain of a nuclear hormone receptor. Further characterization of these unique *C. irritans *proteins could lead to the identification of potential markers for *C. irritans *detection. Thus, the comparative genomics analysis performed in this study provided an overview of conserved gene families in protozoans.

**Table 3:** The summary of SUPERFAMILY and Pfam domains of C *. irritan s*, *T. thermophila *and *P. falciparum*

	*C. irritans*	*T. thermophila*	*P. falciparum*
Total peptide sequences	2 659	24 725	5460
Sequences with SUPERFAMILY domain assignments	1 025(39%)	11268(46%)	2468(45%)
Total SUPERFAMILY hits	1 163	17379	3553
Unique SUPERFAMILY domains	323	715	1101
Sequences with Pfam domain assignments	1 043(39%)	13 896 (56%)	2492 (45%)
Total Pfam hits	1 203	39507	5542
Unique Pfam domains	608	4 168	1 101

### SSR motif analysis

Mining of the EST data for SSRs identified a total of 317 UTs containing 375 nonredundant SSRs. Motifs containing 10, 6, and 5 repeat units of mononucleotides, dinucleotides and higher-order repeats, respectively, were considered to be major microsatellites. A total of 30 UTs contained more than one SSR. The nonredundant EST-derived SSRs were composed of mono-, di-, tri- and tetranucleotide repeat motifs only although motifs containing repeated units of 1-6 nucleotides in length were considered SSRs and were searched by MISA. The frequency of the SSR motifs identified in the 317 UTs is summarized in Additional File 4. The distribution of SSR motifs revealed the presence of A/T homopolymers in up to 76% of the total SSRs. This might be due to the A/T-rich content of the ciliate genome and transcriptome [47]. The AC/GT and AT/AT dinucleotide SSR motifs were present in equal numbers and accounted for 9% of the SSRs identified. AAT/ATT was the most widespread trinucleotide among the nine trinucleotide SSR motifs present in the UTs. Only the AAAC/GTTT, AAAT/ATTT and AACT/ATTG tetranucleotide SSR motifs were present in the UTs and each occurred only once. *C. irritans *shows intraspecific variation; therefore, these SSRs within ESTs could serve as microsatellite markers for variant discrimination, geographical differentiation, mixed infection identification and also for lineage and population studies of this parasite [48]. Microsatellites have also been used for the detection of drug-resistant variants of parasites [49]. Screening of ESTs is known to be a cost-effective and efficient method for detecting utilizable microsatellite markers [49].

### Functional annotation

#### Gene Ontology annotation

**Figure 5:**
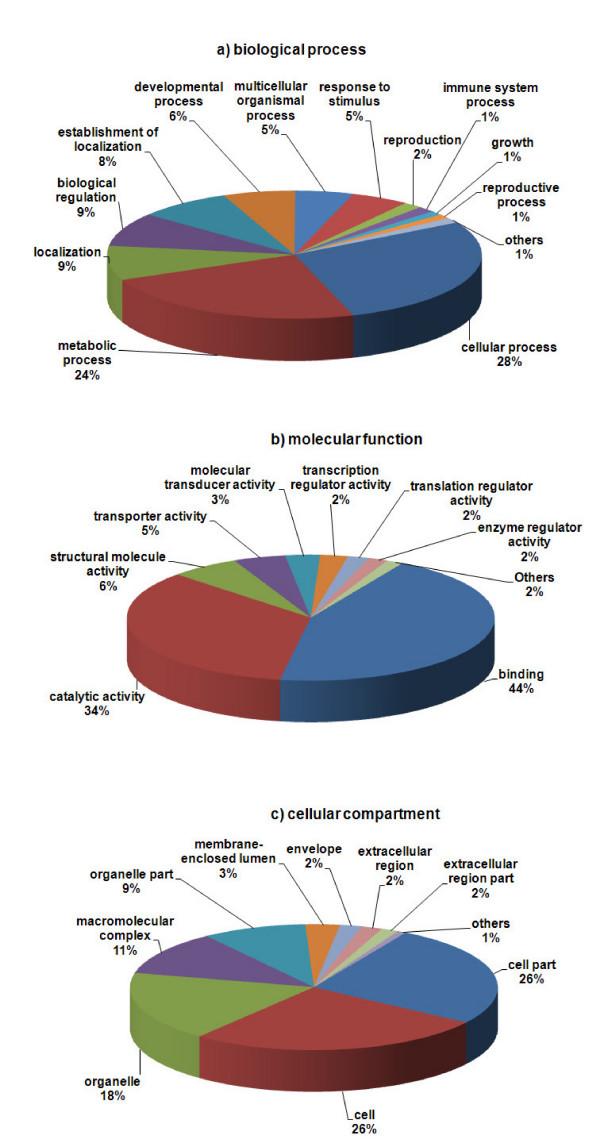
**GO annotation**. GO annotation was done with the Blast2GO tool. The level 2 GO terms which are (a) biological process, (b) molecular function, and (c) cellular component are shown.

As in the case of other ciliates, *C. irritans *also uses TAA and TAG as glutamine codons instead of termination codons [50]. The UTs were translated into peptide sequences using the Ciliate, Dasycladacean and Hexamita Nuclear Code, and the longest CDSs were obtained by using options set to begin a CDS without a start codon. The peptide sequence translated from the longest CDS was loaded into Blast2GO and the BLASTP function was run against the GenBank nr protein database. Subsequently, Blast2GO was used to map and annotate GO terms based on the BLASTP results. A total of 790 UTs consisting of 248 consensi and 542 singletons were annotated with 1782 GO terms. The GO terms were distributed into the 3 main GO categories of biological process (601), molecular function (661) and cellular component (520). The remaining UTs were not annotated due to any one of the following reasons: they did not result in a BLASTP hit, were not successfully mapped, or were not annotated after mapping because the UTs failed to fulfill the annotation criteria. The GO distribution charts by 2^nd ^level GO terms are shown in Figure 5, and the complete GO annotation findings are provided in Additional File 1. Cellular process (27%) and metabolic process (24%) were the main subcategories of biological process. This was expected because the tomont is the dividing stage, which involves the cell cycle as well as the generation and use of energy. GO annotation under biological process showed the presence of many classes of proteins important for pathogenesis and continuous generation of the parasite such as those involved in cell adhesion, stress response, circadian rhythm, dormancy process, death, cell communication and secretion. The molecular function category was mainly comprised of proteins involved in binding (45%) and catalytic activities (34%).

Subcategorization of this category also led to the identification of various groups of proteins that can be exploited to control this parasite, mainly by using inhibitors of the proteins involved in cytoskeletal protein binding, proteins with hydrolase activity, and proteins with transferase activity. The cellular compartment consists of the following subcomponents: cell parts (27%), cell (26%), and organelles (18%). Proteins that were annotated as the external encapsulating structure, cell projection proteins, and proteinaceous extracellular matrix under the GO category cellular component were those with potential as serodiagnostic markers of the tomont stage parasites. An InterProScan was performed via Blast2GO returned hits on 1273 UTs, which included 77 UTs with no previous significant hits and 77 UTs that were similar to hypothetical proteins in the nr protein database of NCBI (Additional File 1).

#### KEGG pathway assignment

KAAS annotated a total of 746 UTs with KO terms, and these were further classified into 40 pathways containing 382 UTs by using the pathway identification tool in KOBAS. The top 10 pathways are summarized in Table 4. Ribosomal proteins (47 UTs) accounted for the highest number of proteins mapped to the KEGG pathways. Other KEGG pathways with a high number of UTs were those for chaperones and folding catalysts, cell cycle events, and oxidative phosphorylation with 28, 26 and 22 UTs, respectively. We also found three UTs that mapped to the Jak-STAT signaling pathway, whereas *T. thermophila *genes that are involved in this pathway are yet to be identified or do not exist. Various genes that are involved in the cell cycle and apoptosis pathways were also identified, and these can be further studied for future therapeutic strategies and control applications for *C. irritans*.

**Table 4:** Top 30 metabolic pathways in *C. irritans *mapped by KEGG

No.	KEGG pathway	No. UTs	Background genes distribution^a^
1	Ribosome	47	69
2	Chaperones and folding catalysts	28	57
3	Cell cycle	26	24
4	Oxidative phosphorylation	22	49
5	Translation factors	19	41
6	Pyruvate metabolism	18	36
7	Transcription factors	15	3
8	Cell cycle - yeast	14	24
9	Insulin signaling pathway	14	27
10	Proteasome	13	27
11	Apoptosis	12	10
12	Wnt signaling pathway	11	14
13	Focal adhesion	10	5
14	Prostate cancer	10	9
15	Regulation of actin cytoskeleton	10	13
16	Beta-alanine metabolism	9	12
17	Epithelial cell signaling in *Helicobacter pylori *infection	9	14
18	Receptors and channels	8	2
19	Long-term potentiation	8	8
20	Tight junction	8	9
21	Melanogenesis	7	5
22	p53 signaling pathway	7	10
23	Huntington's disease	6	4
24	TGF-beta signaling pathway	6	8
25	Notch signaling pathway	5	2
26	Adherence junction	5	4
27	Thyroid cancer	4	2
28	Renal cell carcinoma	4	3
29	Jak-STAT signaling pathway	3	0
30	Neuroactive ligand-receptor interaction	3	1

^**a **^Number of genes present for the particular pathway in whole gene data set of *Tetrahymena thermophila*.

### Prediction of potential proteins that have potential use as diagnostic markers and vaccine candidates

#### Membrane protein prediction

Membrane proteins were predicted by identifying the transmembrane region and signal peptide. Most of the methods used for predicting membrane proteins do not discriminate well between signal peptides and membrane-spanning regions [51]; therefore, all peptides with a single transmembrane region that overlapped a signal peptide were not regarded as transmembrane proteins. All sequences predicted to contain more than one transmembrane region or contain single transmembrane regions that do not overlap with a signal peptide or signal anchor region were predicted to be membrane protein coding genes. Peptides with only a signal anchor or a signal anchor that overlaps with a sole transmembrane region were considered to be membrane-bound proteins. A total of 481 membrane proteins and 54 membrane bound proteins were predicted (Additional File 1). Among the 481 predicted membrane proteins, 309 were predicted to contain more than one transmembrane region. In addition, in the GPI-anchor prediction analysis showed only two peptides that were identified as GPI-anchored proteins by all three prediction tools. It is noteworthy that GPI-SOM, which classifies GPI-anchored proteins by detecting both the N-terminal signal peptide and C-terminal GPI-anchor signal, identified 39 peptides as GPI-anchored proteins. A total of 73 peptide sequences were found to contain a GPI-anchor signal by at least one of the three tools (Additional File 1).

#### Excretory/Secretory protein prediction

Excretory/secretory proteins (ESPs) of parasites enable these organisms to invade and parasitize the host cell. The ESPs can be used as immunodiagnostic, drug and vaccine candidates because several studies have shown that antibodies against ESPs protect or reduce parasite infection [52,53]. Peptide sequences that were predicted by SignalP [31] to contain a signal peptide but not contain any transmembrane regions (as predicted by Localizome [32], ProtCOMP 6.1 [33] and TMHMM 2.0 [34]), were classified as ESPs. A total of 155 UTs were predicted to be ESPs. Of these, 64 (41.3%) UTs had no significant similarity to any of the protein sequences publicly available, and another 10 UTs had matches with hypothetical proteins or proteins of unknown function. A total of 43 (27.7%) ESPs were homologs of ciliate proteins (Additional File 1). One group of ESPs that was found to be highly expressed (8% of ESPs) contained members of the cysteine protease family such as calpain, papain, and cathepsin. These proteolytic enzymes are known to be involved in host cell invasion, encystation, excystation, catabolism of host proteins, differentiation, cell cycle progression, cytoadherence, and evasion of host immune responses [54]. Cysteine proteases, which are strongly immunogenic, are potential as vaccine candidates, therapeutic targets, and also serodiagnostic markers of parasites [55,56]. Therefore, these highly expressed cysteine proteases can be exploited for the detection of *C. irritans *in water and can also serve as therapeutic targets of selective protease inhibitors [55]. Another interesting finding was the identification of leishmanolysin domain-containing proteases, which were identified as ESPs. Leishmanolysin is a GPI-anchored surface protein originally identified as a virulence factor of *Leishmania major*. However, later, it was also found in ciliates such as *T. thermophila *[57]. The prediction of leishmanolysin as an ESP in this data sets may be due to the partial sequencing of the UTs that might have hindered the identification of the C-terminal GPI-anchor. Another highly expressed ESP in *C. irritans *was the disulfide-isomerase domain-containing protein; it is required for catalyzing disulfide bond formation and is also a target for inhibitors [58]. BNR/Asp-box repeat family proteins are also major secreted ESPs in the *C. irritans *tomont stage. The functions of these proteins remain to be determined, although BNR/Asp box repeats are mainly found in glycosyl hydrolases such as sialidases and in other secreted proteins [59].

#### Peptide repeats analysis

Repeats are widely found in disease-causing parasites such as *P. falciparum*, *Trypanosoma brucei*, and *L. major *[40]. We used the RepSeq and Reptile tools to identify repeats in the translated UTs. Although RepSeq functions with any given proteome, it is designed for repeat analysis in lower eukaryotic pathogens [40] and is therefore well-suited for repeat analysis in *C. irritans*. Motifs containing 10, 3, and 2 repeat units of a single amino acid, tandem repeats (di-amino acid), and sequence repeat regions (SSRs, 6-amino acid), respectively, were considered to be major peptide repeats. The type and number of UTs containing these repeats are presented in Additional File 4. Reptile was also used to predict these repeats because the RepSeq tool only searches for single, double and 6-amino acid repetitions and misses all other repetitions. Reptile found a total of 373 UTs consisting of 101 consensi and 276 singletons that contained repeats. Most of the repeats were repetitions of single or double amino acid motifs. The proteins that were found to contain repetitive motifs were further studied to determine their localization and putative functions. The results are summarized in Table 5. These proteins should be further studied as potential diagnostic markers for *C. irritans *infection in mariculture systems and as vaccine candidates based on their localization and the presence of repetitive motifs [39]. However, these proteins were identified from the non-infective stage, and their presence in the infective stage of the parasite needs to be confirmed prior to further studies on their use in vaccines.

**Table 5:** Repetitive motifs containing transmembrane and extracellular proteins

ID	BLASTX	Pfam/Interpro	L	Repeat		ESTs
cn349	Multicystatin	No significant hit	EC	2X	DKIPKSVLEFGINKLELSNVFAHKDFSKIENAQMKVVSGYIYKFTLVYQFSEQEHKFEIQVWSKADQTLELISMKEIT	7
cn66	Unknown protein	EGF-like domain	EC	4X	YVNNGSCSSNSTLFNFTSKNCEKSCG	22
cn65	Insect antifreeze protein	EGF-like domain	EC	5X	LFNFTSKNCEKSCGESGY	10
cn131	Protein disulfide-isomerase domain containing protein	Protein disulphide isomerase family	EC	3X	EEKEEK	9
cn85	Predicted protein	Ricin-type beta-trefoil lectin domain	EC	4X	VLDVYG	8
cn81	Chitinase	Glycoside hydrolase, family 18 domain, chitinase active site	EC	2X	YARGYELCKTPGDKLDKIFYAFLNPTTG	
CiTo 53E07	Membrane associated protein	No significant hit	TM	9X	FSFLLFFFFFSFWS	1
CiTo 12F11	No significant hit	EGF-like domain, Metridin-like ShK toxin	EC	3X	AETGST	1
CiTo 13H10	Tenascin	EGF domain	TM	3X	PNNCSG	
CiTo 26C11	No significant hit	No significant hit	TM	3X	EKCRCL	1
CiTo 2H11	No significant hit	No significant hit	EC	3X	AKTAAE	1
CiTo 6G09	Cation diffusion facilitator family transporter	Cation efflux protein family	TM	3X	GHGHSH	1

L, Localization; TM, transmembrane domain; EC, extracellular

## Discussion

Tomonts represent an important stage in the life cycle of *C. irritans *because they ensure the continuity of the parasite by releasing asynchronous theronts from day 3 to day 35 post-encystment, even though they are incubated under similar conditions [2]. This is a serious obstacle in total eradication of the parasite because tomonts are resistant to most of the chemotherapeutics tested so far when these are administered at a dose that is nontoxic to the fish [2]. In addition, at present, there is no reliable *in vitro *culture method available for continuously propagating *C. irritans *in a host-free system [60]. Selection of the tomont stage, which is external to the host and sediments at the bottom of the aquarium, facilitated the collection of sufficient amounts of sample for this study. Using the tomont stage *C. irritans *samples, we successfully constructed a high-quality cDNA library with a recombination efficiency rate of 93% and titer of 1.28 × 10^6 ^pfu. The assembly of 5356 EST sequences aided the identification of 2659 UTs. These data provide a useful functional genomics resource for this economically important fish parasite. The transcriptomic data of the *C. irritans *tomont stage have led to gene discovery and provided an insight into the genomics of the parasite. Future studies on the expression profile of *C. irritans *at other stages of the life cycle will facilitate the identification and differentiation of genes involved in all stages of the life cycle versus those involved only in certain stages of the life cycle. Moreover, this could provide an insight into the stage-specific functions of *C. irritans *and the genes involved in the pathogenesis of this parasite.

Phylogenetic comparison of the *C. irritans *β-tubulin amino acid sequence supported earlier findings that the parasitic ciliate *C. irritans *is taxonomically distinct from the fresh-water parasitic ciliate *I. multifiliis*. This justified the classification of *C. irritans *under a different class within the phylum Ciliophora (Class: Prostomatea) despite the striking common features and parallel life cycles of the two parasites [1,43]. The distinction between *C. irritans *and *I. multifiliis *further supports the failure to detect any genes unique to *C. irritans *and *I. multifiliis *based on comparison of their currently available EST datasets. The absence of solely shared genes between these parasites and their distant phylogenetic relationship showed that the mechanism and molecules involved in their life cycle and pathogenicity differed considerably. These transcriptomic and taxonomic data also demonstrate that their parasitic lifestyles have evolved independently, confirming previous reports that the common features of these two parasites are due to adaptive convergence rather than evolutionary relatedness [1,45].

The ESTs of ribosomal and mitochondrial proteins, which are normally removed during normalization or preprocessing of ESTs, were not removed in this study. A survey of existing literature shows that the levels of ribosomal protein gene expression differ at different stages of the life cycle. In addition to protein biosynthesis, ribosomal proteins play various roles, termed extra-ribosomal functions, which include transcription, signal recognition, apoptosis, and nuclear transport protein synthesis [61,62]. Therefore, the UTs encoding ribosomal and mitochondrial proteins should complement ESTs from the other stages of the parasite life cycle as this would help in obtaining a better understanding of their stage-specific functions.

Many of the potential genes identified at the tomont stage in this study for the diagnosis and control of *C. irritans *are also expected to be expressed at other stages of the *C. irritans *life cycle. These proteins should facilitate the design of non-stage-specific control and diagnostic methods to overcome the difficulties in eradicating *C. irritans *due to asynchronous theront release from tomonts and asynchronous trophont exit from the host. Development of a vaccine, however, requires additional studies to ensure that the selected antigen is present in the theront stage. This would increase the probability of the antigen conferring immunity to the host against the infective stage of *C. irritans*.

One protein that has been much studied in *C. irritans *is the agglutination/immobilization antigen [50,63]. This protein is regarded as the *C. irritans *immobilization antigen (i-antigen) [50]. The i-antigens in other ciliates such as *T. thermophila*, *Paramecium aurelia*, and *I. multifiliis *and the protective immunity provided by antibodies produced against i-antigens have been reported previously [64]. It is also known that this protein is expressed in various isoforms and serotype variants in *C. irritans *(GenBank AB262047--AB262051; [50,63]). Agglutination/immobilization antigen isoform 1 was reported to be present in both the theront and trophont stages of *C. irritans *and this antigen is predicted to be expressed in the cilia of the parasite [50]. Although the function of the protein is unknown, it is abundantly expressed in the tomont stage of *C. irritans*. A total of six UTs (including cn41, cn42, and cn57 (Table 2)) were similar to agglutination/immobilization antigen isoform 1, while three other UTs were similar to agglutination/immobilization antigen isoform 4. However, at the protein level, there is only 41%-71% similarity between the UTs in this study and previously reported agglutination/immobilization antigen isoforms. Use of ClustalW 2.0 for multiple sequence alignment of the translated nucleotide query of all nine UTs with all i-antigens sequences available in the GenBank nr protein database showed that the 12 cysteine residues are conserved in all but one of the sequences. Thus, it is presumed that UTs with agglutination/immobilization antigen features have similar structures. Variants of these transcripts with possibly similar functions might have arisen as a result of the presence of various *C. irritans *serotypes within the environment or due to a gene duplication event in which the parasite might have expanded the members of the gene family as a response to environmental changes or as a survival strategy [63,65,66]. Most probably, alternative splicing did not lead to the creation of the these variants because alternative splicing is uncommon in ciliates [67]. Its occurrence was also not supported by the multiple sequence alignment data (data not shown). The agglutination/immobilization antigen is a potential vaccine candidate for white spot disease because it is expressed at both the theront and tomont stages [[38]; this study]. However, the serotype-specific protection conferred upon the fish by agglutination/immobilization antigens as shown by Hatanaka (2008) [63] and the existence of various isoforms are some obstacles that need to be overcome before this protein can be developed and used as a vaccine against *C. irritans*. In addition to the agglutination/immobilization antigen, several other genes encoding potential vaccine candidates and targets for detection and therapeutic applications were identified in this EST study. Among these were the genes encoding predicted surface proteins, GPI-anchored proteins, ESPs, and proteins with repetitive amino acids. Various studies have been undertaken on apicomplexan parasites such as *Plasmodium vivax, Toxoplasma gondii*, and *Trypanosoma brucei *to identify surface proteins, examine their role in pathogenicity, and determine their potential as vaccine candidates [68,69]. Many putative membrane proteins identified in this study have significant similarity to transporter proteins that are integral membrane proteins involved in the transport of molecules across biological membranes. Transporter proteins are also found to confer protection against bacterial infections and have also been extensively studied in drug-resistant parasitic protozoa [70]. GPI-anchored proteins have also been widely studied as vaccine candidates in parasitic protozoa including *C. irritans*[63] because these proteins are common on the surface of protozoan parasites and are involved in stimulating or inhibiting various host immunological responses [71].

ESPs are involved in molecular interactions with host cells and are exposed to the host immune system; therefore, these could also act as protective antigens and represent potential vaccine candidates as well as serodiagnostic molecules [52,53]. Moreover, inhibition of essential ESPs could prevent invasion and growth of the parasite [55]. The *C. irritans *proteases identified in this study could be good targets for further studies on protease inhibition by various inhibitors [55].

Proteins with repeated amino acid motifs are implicated in antigenic diversity and recognition, host-cell receptor binding and stimulation of the host immune response [40]. Repeat-containing proteins such as the *P. falciparum *histidine-rich protein-2 (*Pf *HRP2) are also being studied as potential diagnostic markers [72]. However, antigenic polymorphisms facilitate the evasion of host immune responses elicited by past exposure to the same antigen, which leads to difficulties in the development of repeat-containing antigens as vaccines [73].

Another group of ESTs that were identified in this study and could be useful are the enzymes and proteins involved in cyst wall synthesis and differentiation. Since the ESTs were generated from the cyst stage, enzymes and other proteins involved in cyst wall synthesis and differentiation, such as the chitin synthase family proteins, UDP-glucose 4-epimerase family proteins, and UDP-glucose/GDP-mannose dehydrogenase family proteins, were identified in the EST data set. Disruption of cyst wall synthesis, differentiation and integrity by using chemotherapeutic agents may prevent encystment into tomonts.

Previous studies with *C. irritans *showed that codons that encode stop signals in standard translation systems are used to encode glutamine in this organism. This is also the case in other ciliates [50,74]. This was further confirmed in this study in which the TAA and TAG codons appeared in most of the ESTs. Moreover, use of the ciliate translation code in the Virtual Ribosome tool resulted in longer CDSs, whereas use of the standard translation code resulted in unreasonably short CDSs. Thus, the nonstandard translation system of ciliates requires additional research before any protein of interest is expressed because the expression of ciliate proteins in common expression systems using *Escherichia coli *or yeast will result in premature polypeptide chain termination. This has been a major complication in conducting various studies that require the expression of the targeted protein. Although expression in *E. coli *with suppressor tRNA-encoding expression vectors or site-directed mutagenesis is possible, such procedures are laborious and costly. Moreover, they may not be applicable to all proteins and generally meet with limited success [75]. The expression of the *I. multifiliis *surface protein in *T. thermophila *is promising [76], but the unavailability of a commercial ciliate expression vector and transformation host as well as the special transformation method required (DNA bombardment) may hinder routine ciliate expression studies. However, synthetic genes offer an alternative for heterologous protein expression in common expression systems [77]. This technology in combination with the availability of potential genes for the control of *C. irritans *identified in this EST study should allow the expression of *C. irritans *proteins for drug screening, vaccine trials, and diagnostic tests.

## Conclusions

In this study, we report the first ever *C. irritans *transcriptome data set of 5356 high-quality ESTs consisting of 2659 UTs. The results provide new insights into the genomics of this aquaculture parasite. Approximately 26% (693) of the UTs were identified to be novel sequences, while 57% were found to be similar to ciliate sequences. We also identified UTs that encode various potential *C. irritans *diagnostic and therapeutic candidates. These should be useful in developing *C. irritans *diagnostic and control strategies via molecular techniques.

## Authors' contributions

YL collected the samples, constructed the library, sequenced the clones, analyzed and interpreted the data, and drafted the manuscript. SN and AMA conceived and designed the study and interpreted the data. They also participated in drafting the manuscript. K-LW participated in revising the manuscript. All authors have read and approved the final manuscript.

## Supplementary Material

Additional file 1 **BLASTX result, GO annotation, InterProScan result and prediction of potential genes for usage in *C. irritans *diagnostic and control strategy**. The BLASTX results against the non-redundant protein database of NCBI, the GO annotations of the UTs and InterProScan results as performed by the Blast2Go tool, transmembrane regions, GPI-anchor signal and signal peptide prediction. Click here for file

Additional file 2 **Comparison of BLASTX results against the non-redundant protein database of NCBI, *T. thermophila *and *P. falciparum***. The BLASTX results against the non-redundant protein database of NCBI, *Tetrahymena thermophila *and *Plasmodium falciparum*. Click here for file

Additional file 3 **SUPERFAMILY and Pfam domains of *C. irritans*, *T. thermophila *and *P. falciparum***. Number of protein domain assignments of *C. irritans*, *T. thermophila *and *P. falciparum *according to SUPERFAMILY and Pfam classifications. Click here for file

Additional file 4 **Nucleotide and protein repeats summary**. The The frequency of SSR motifs in the *C. irritans *UTs and number of amino acid repeats identified in the translated UTs by RepSeq tool. Click here for file
